# Earthworms drastically change fungal and bacterial communities during vermicomposting of sewage sludge

**DOI:** 10.1038/s41598-021-95099-z

**Published:** 2021-07-30

**Authors:** Jorge Domínguez, Manuel Aira, Keith A. Crandall, Marcos Pérez-Losada

**Affiliations:** 1 Grupo de Ecoloxía Animal (GEA), Universidade de Vigo, 36310 Vigo, Spain; 2 Department of Biostatistics and Bioinformatics, Computational Biology Institute, Milken Institute School of Public Health, George Washington University, Washington, DC 20052 USA; 3 CIBIO-InBIO, Centro de Investigação em Biodiversidade e Recursos Genéticos, Universidade do Porto, Campus Agrário de Vairão, 4485-661 Vairão, Portugal

**Keywords:** Environmental sciences, Microbial communities, Environmental microbiology, Microbial ecology

## Abstract

Wastewater treatment plants produce hundreds of million tons of sewage sludge every year all over the world. Vermicomposting is well established worldwide and has been successful at processing sewage sludge, which can contribute to alleviate the severe environmental problems caused by its disposal. Here, we utilized 16S and ITS rRNA high-throughput sequencing to characterize bacterial and fungal community composition and structure during the gut- and cast-associated processes (GAP and CAP, respectively) of vermicomposting of sewage sludge. Bacterial and fungal communities of earthworm casts were mainly composed of microbial taxa not found in the sewage sludge; thus most of the bacterial (96%) and fungal (91%) taxa in the sewage sludge were eliminated during vermicomposting, mainly through the GAP. Upon completion of GAP and during CAP, modified microbial communities undergo a succession process leading to more diverse microbiotas than those found in sewage sludge. Consequently, bacterial and fungal community composition changed significantly during vermicomposting. Vermicomposting of sewage resulted in a stable and rich microbial community with potential biostimulant properties that may aid plant growth. Our results support the use of vermicompost derived from sewage sludge for sustainable agricultural practices, if heavy metals or other pollutants are under legislation limits or adequately treated.

## Introduction

The amount of sewage sludge generated in wastewater treatment plants (WWTPs) keeps steadily increasing and hundreds of million tons are produced every year all over the world. In Europe, nearly 50% of this sludge is dumped in agriculture and forestry soils, with less than 25% being effectively composted or recycled (http://ec.europa.eu/eurostat). The disposal of sewage biosolids may cause severe environmental problems, particularly in terms of soil pollution by heavy metals, human pathogens and organic pollutants, including new emerging contaminants.

European Union (EU) waste management policies promote recycling processes and discourage landfill disposal. Currently, Council Directive 199/31/EC of 26 April 1999 on the landfill of waste, and their later amendments stablish that Member States shall set up a national strategy for the implementation of the reduction of biodegradable waste going to landfills. It also obliges Member States to reduce biodegradable municipal waste going to landfills to 35% of the total amount (by weight) of biodegradable municipal waste produced and directs them to different value-forming processes focused mainly on its use as organic fertilizers because of its nutrient content. The EU Strategy for Circular Economy also endorses this policy and has set the limit of landfilling to 10% of the current volume of dumping into landfills by 2035. This EU strategy also considers recycled nutrients as an important category of secondary raw materials. In the case of organic wastes, their treatment and further use in agriculture contribute to reduce the use of mineral-based fertilizers and consumption of limited non-renewable resources such as phosphate rocks. However, these wastes must be free of hazardous substances in order to be safely used in agriculture.

Vermicomposting is an enhanced biooxidative process in which detritivorous earthworms (mainly *Eisenia* spp.) and microorganisms acting together accelerate the decomposition process and modify critically the physical, chemical and biological properties of organic wastes^1–4^. Vermicomposting is well established worldwide and has already been successful in processing sewage sludge, reducing the content of microbial pathogens and the bioavailability of heavy metals^5–8^.

Vermicomposting involves an active phase, where earthworm activity is critical, and a maturation phase, which takes place once worms leave the substrate, and where microorganisms take control and are the key players. The active phase comprises all the processes associated with the passage of substrate through the earthworm intestines (GAPs: gut-associated processes)^2,3^. During this phase, earthworm digestion reduces microbial biomass and activity and modifies the structure and function of the microbial communities during the vermicomposting process^9–12^. In the maturation phase, earthworm excreted materials or casts start aging, while their associated microbial communities experience a turnover (i.e., cast-associated processes, CAPs)^2,4,13,14^.

During GAP and CAP, earthworms destroy pathogenic microorganisms, but species-specific elimination rates and effectiveness are poorly understood^15^. On the other hand, vermicompost has good physical, chemical and biological properties, including particular microbiomes, which provide a whole range of beneficial effects to the soil–plant system^16^. Those effects have been found to be independent of the chemical composition of the vermicompost, hence suggesting they are likely related to biostimulation mechanisms derived from microbial activity^17,18^. Since vermicompost is an organic biofertilizer that is of interest not only for its chemical properties and nutrients, but also for its biological properties in terms of microbial inoculums, it is important to thoroughly characterize the structure and composition of its microbial content; particularly of the fungal communities, which have been largely ignored in vermicomposting microbiome research.

Towards that goal, here we have coupled 16S and ITS rRNA high-throughput sequencing and sophisticated metataxonomic analysis to assess the impact of earthworms on the composition and structure of bacterial and fungal communities (microbiomes) during vermicomposting of sewage sludge in relation to GAP and CAP processes.

## Results and discussion

### The composition of bacterial and fungal microbiotas changes during vermicomposting of sewage sludge

**Figure 1:**
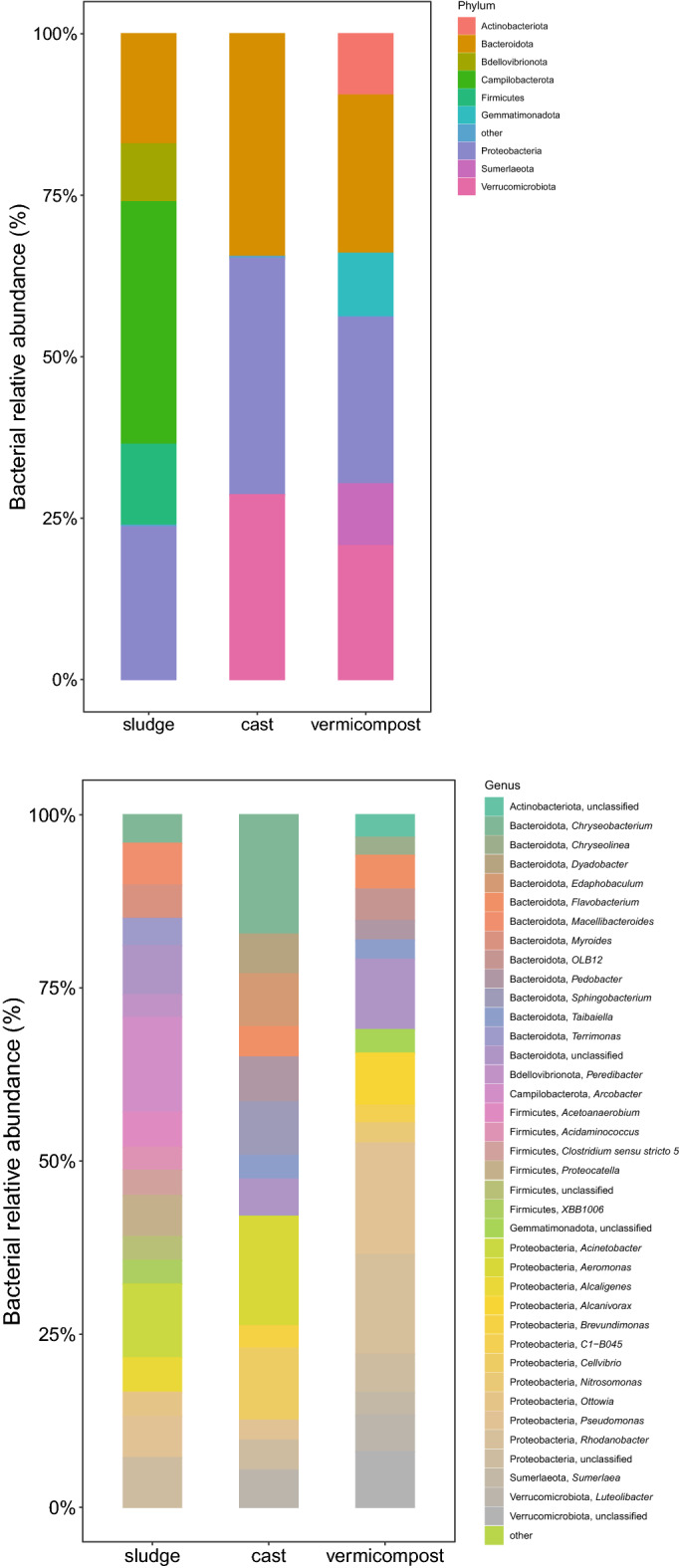
Relative abundance of the main phyla and genera of bacteria in sewage sludge, fresh earthworm casts and vermicompost (3 months old) during vermicomposting of sewage sludge. Low abundance bacterial phyla and genera (< 1%) were grouped together (other).

The bacterial community of the raw sewage sludge included 19 phyla and was mainly comprised of Bacteroidota, Bdellovibrionota, Campilobacterota, Firmicutes and Proteobacteria (Fig. 1). Bacterial communities of fresh earthworm casts were dominated by the phyla Bacteroidota, Proteobacteria and Verrucomicrobiota (Fig. 1). Large changes in bacterial community composition were found after transit of the sewage sludge through the gut of the earthworms (GAP), with significant decreases in the abundance of Campilobacterota, Firmicutes and Bacteroidota, and significant increases in the abundance of Verrucomicrobiota, Proteobacteria and Bacteroidota (Supplementary Table S1). At the genus level, transit through the gut significantly reduced the abundance of bacterial genera *Terrimonas*, *Acetoanaerobium*, *Bacteroides*, *Cloacibacterium*, *Proteocatella* and *Macellibacteroides* among others (Fig. 1, Supplementary Table S2), and increased significantly the abundance of *Dyadobacter*, *Aeromonas*, *Luteolibacter*, *Edaphobaculum*, Cellvibrio, *Pedobacter*, *Sphingomonas*, *Devosia*, *Cetobacterium* and *Rhodanobacter* among others (Fig. 1, Supplementary Table S2). At ASV level, transit through the earthworm gut significantly reduced the relative abundance of 49 bacterial ASVs and increased the relative abundance of 54 bacterial ASVs (Supplementary Table S3).

The bacterial community of the vermicompost was dominated by the phyla Actinobacteria, Bacteroidota, Gemmatimonadota, Proteobacteria, Sumerlaeota and Verrucomicrobiota (Fig. 1). The cast-associated processes (CAP) of the vermicomposting significantly increased the abundance of Actinobacteriota, Gemmatimonadota and Sumerlaeota, and reduced the abundance of Verrucomicrobiota, Bacteroidota and Proteiobacteria (Fig. 1, Supplementary Table S1). At the genus level, CAP processes of vermicomposting resulted in the increase of the abundance of the bacterial genera *Alcanivorax*, *Sumerlaea*, *Arenibacter*, *Nitrosomonas* among others, and significantly reduced the abundance of the bacterial genera *Dyadobacter*, *Aeromonas*, *Brevundimonas*, *Chryseobacterium*, *Sphingomonas*, *Acinetobacter* and *Devosia* among others (Fig. 1, Supplementary Table S2). At ASV level, CAP processes of vermicomposting significantly increased the relative abundance of 40 ASVs and reduced the relative abundance of 15 ASVs (Supplementary Table S3).

**Figure 2:**
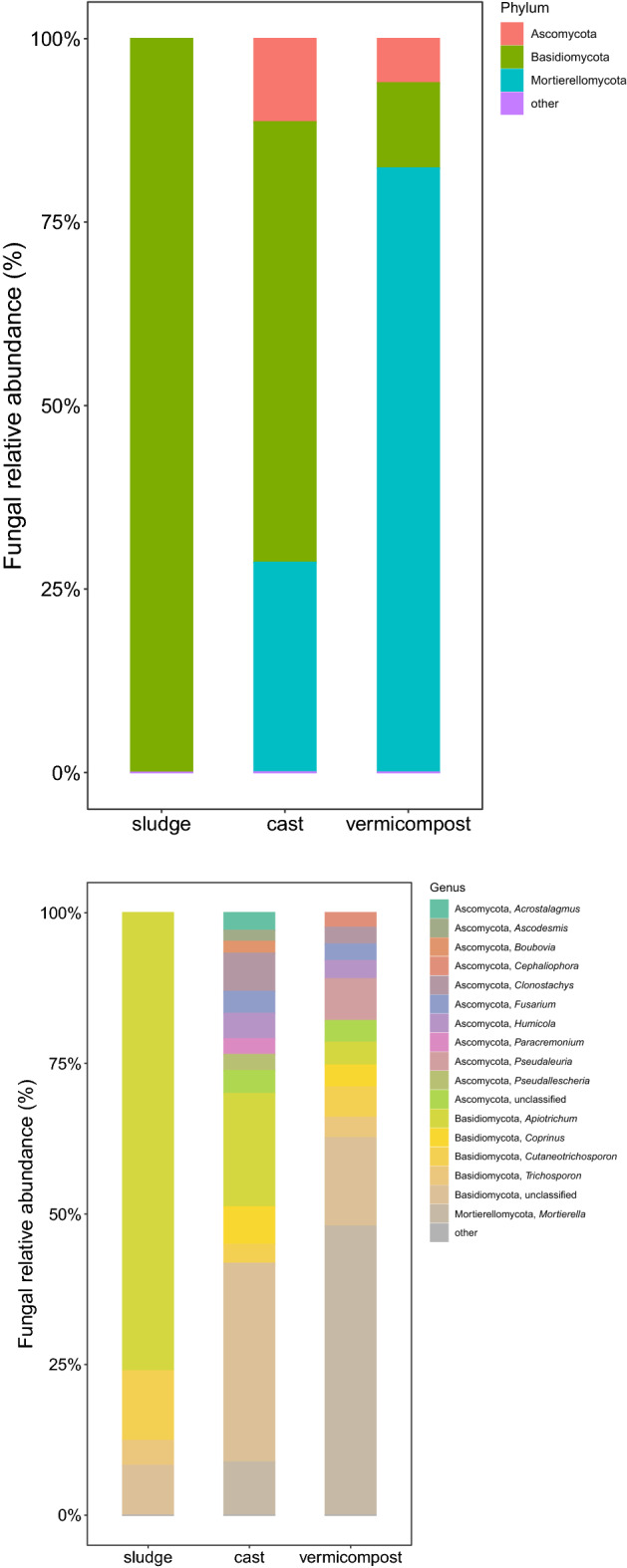
Relative abundance of the main phyla and genera of fungi in sewage sludge, fresh earthworm casts and vermicompost (3 months old) during vermicomposting of sewage sludge. Low abundance bacterial phyla and genera (< 1%) were grouped together (other).

The fungal community of the sewage sludge was almost exclusively dominated by the phylum Basidiomycota (Fig. 2). Large changes in fungal community composition were found after transit of the sewage sludge through the gut of the earthworms (GAP), with significant decreases in the abundance of Basidiomycota and significant increases in the abundance of Ascomycota and Mortierellomycota (Fig. 2, Supplementary Table S1).

At the genus level, transit through the gut significantly decreased the abundance of *Apiotrichum*, *Candida*, *Kazachstania* and *Trichosporon* (Fig. 2, Supplementary Table S2) and increased significantly the abundance of the fungal genera *Clonostachys*, *Fusarium*, *Coprinus*, *Humicola*, *Paracremonium* and *Mortierella* among others (Fig. 2, Supplementary Table S2). At ASV level, transit through the gut significantly reduced the relative abundance of 25 fungal ASVs and increased the relative abundance of 105 bacterial ASVs (Supplementary Table S3).

The fungal community of the vermicompost was composed of the same phyla as the fresh casts. CAP processes of vermicomposting significantly increased the abundance of the phyla Blastocladiomycota and Mortierellomycota (Fig. 2, Supplementary Table S1). At the genus level, CAP processes of vermicomposting resulted in the increase of the abundance of *Debaryomyces*, *Mortierella*, *Cephaliophora*, *Scedosporium* and *Trichosporon*, and significantly reduced the abundance of *Scutellinia*, *Apiotrichum*, *Paracremonium* and *Boubovia* (Fig. 2, Supplementary Table S2). At ASV level, CAP processes of vermicomposting significantly increased the relative abundance of 74 fungal ASVs and reduced the relative abundance of 91 fungal ASVs (Supplementary Table S3).

Our results partially agree with our previous findings about the vermicomposting of green wastes, where the bacterial composition of the starting materials changed into a vermicompost dominated mainly by Proteobacteria, Bacteroidota, Actinobacteriota and Verrucomicrobiota^12,18–20^. Regarding fungi, few studies have characterized fungal biodiversity in compost and vermicompost; the majority of the most abundant fungal genera found in this study were not described in previous studies^21,22^. As with bacteria, fungal composition of cast and vermicompost was radically different from those of sewage sludge.

These results highlight how deeply vermicomposting modifies bacterial and fungal microbiotas of sewage sludge and demonstrates the critical effect of earthworm gut associated processes. These changes of the microbiota produced by earthworm activity were postulated as the main reasons for the mitigation of antibiotic resistance genes (ARGs) during vermicomposting of sewage sludge^23–25^.

### Bacterial and fungal α- and β-diversity change during vermicomposting of sewage sludge

**Figure 3:**
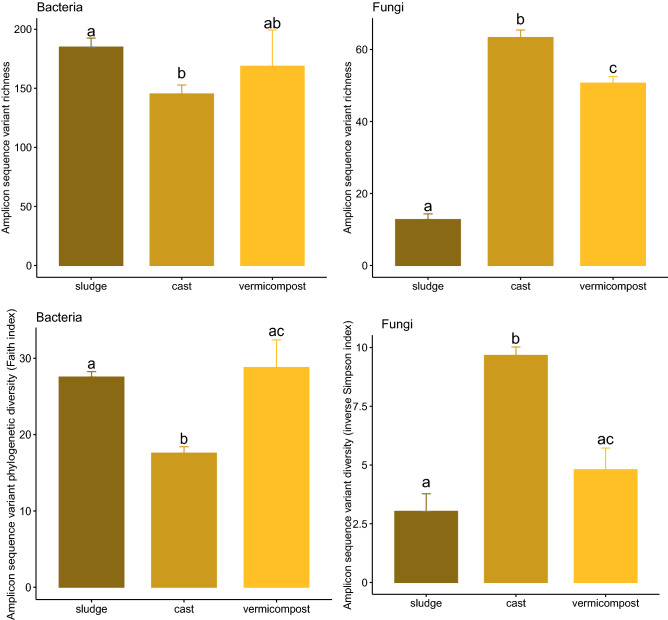
Changes in α-diversity of bacterial and fungal communities during vermicomposting of sewage sludge denoted by observed ASV richness (top) and the Faith and inverse Simpson index (bottom for bacteria and fungi respectively). Different letters indicate significant differences between sewage sludge, fresh earthworm casts and vermicompost (paired Wilcoxon test, FDR corrected).

Bacterial α-diversity decreased moderately in sewage sludge after transit through the earthworm gut (GAP), with significant decreases in ASV richness, Faith phylogenetic diversity (Fig. 3) and Chao1 richness (Supplementary Fig. S1). The cast-associated processes (CAP) of vermicomposting slightly increased bacterial α-diversity, with significant increases in Faith phylogenetic diversity (Fig. 3).

Fungal α-diversity in the sewage sludge increased greatly after transit through the earthworm gut (GAP), with significant increases in fungal ASV richness, inverse Simpson diversity (Fig. 3) and Chao1 richness (Supplementary Fig. S1). The cast-associated processes (CAP) of vermicomposting decreased fungal α-diversity, with significant decreases in richness and diversity (Fig. 3).

Our results disagree with previous findings about the vermicomposting of green wastes, including Scotch broom and different types of grape marc, where bacterial α-diversity increased with vermicomposting^12,18–20,26^. In those studies, the starting material had not previously been processed by an animal gut, and bacterial diversity was low. Here, vermicomposting of sewage sludge, a material already processed by the human gut and therefore more microbially rich, reduced its bacterial diversity due to the earthworm gut-associated processes. These findings indicate that microbial succession during vermicomposting is strongly influenced by the starting substrate. On this regard, and since sewage sludge is highly variable due to its heterogeneous nature and the different methodologies applied in wastewater treatment plants, it would be necessary to verify the performance and magnitude of the vermicomposting process on different types of sludge and or biosolids.

**Figure 4:**
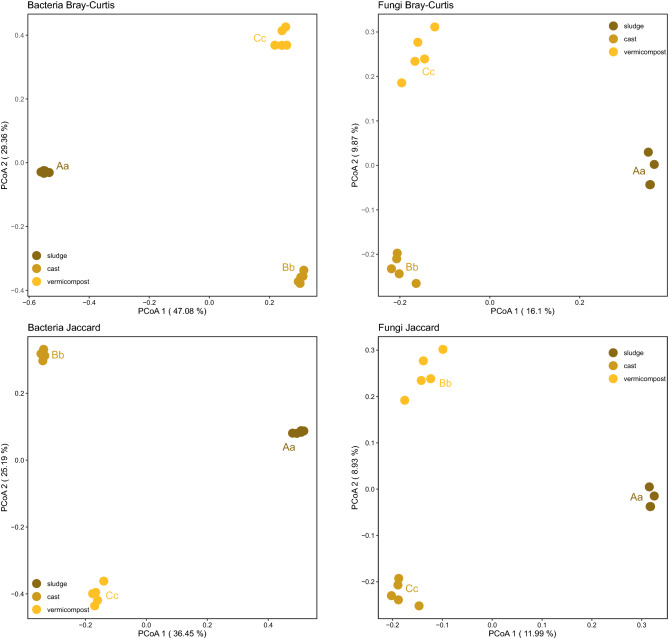
Changes in β-diversity of bacterial and fungal communities during vermicomposting of sewage sludge*.* Principal coordinate analysis with Bray–Curtis and Jaccard distances. Different capital and lower-case letters indicate significant differences between sewage sludge, fresh earthworm casts and vermicompost in PCoA 1 and PCoA 2 scores respectively (paired Wilcoxon test, FDR corrected).

Changes in bacterial and fungal α-diversity of sewage sludge during vermicomposting were accompanied by drastic changes in bacterial and fungal β-diversity during both GAP and CAP associated processes (Fig. 4). Thus, bacterial and fungal communities of sewage sludge, fresh earthworm casts and vermicompost (3 months old) were all significantly different in PCoA 1 and PCoA 2 for Bray–Curtis, Jaccard (Fig. 4) and weighted and unweighted UniFrac distance matrices (Supplementary Fig. S2).

**Figure 5:**
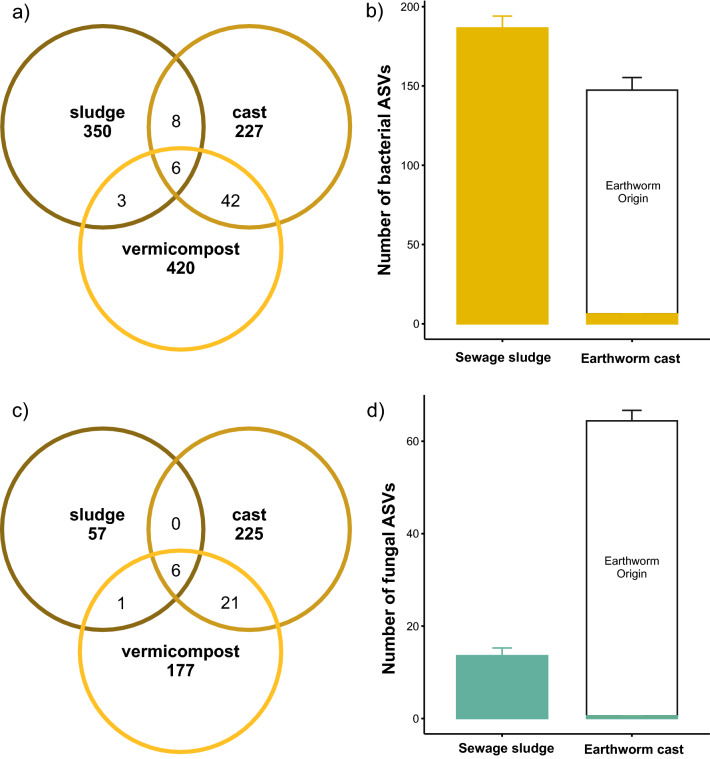
Changes in richness and diversity of bacteria and fungi during vermicomposting of sewage sludge*.* Venn diagrams showing the absolute number of bacterial (**a**) and fungal (**c**) ASVs found in sewage sludge, fresh earthworms casts and vermicompost (3 months old). Effect of GAP (gut-associated processes) on the richness and diversity of (**b**) bacteria and (**d**) fungi.

Only 6 bacterial and fungal ASVs were shared or present in sewage sludge, fresh earthworm casts and vermicompost (Fig. 5a,c, Supplementary Tables S4–S5). This suggests that vermicomposting eliminates 96% of the initial bacterial ASVs and 91% of the initial fungal ASVs as sludge passes through the earthworm gut, the GAP processes of the vermicomposting process (Fig. 5b,d). The CAP processes increased the diversity of the bacterial community and decreased the diversity of the fungal community of the vermicompost derived from sewage sludge (Fig. 5a,c, Supplementary Tables S4, S5). According with the observed differences in β-diversity, bacterial and fungal communities of sewage sludge, fresh earthworm casts and vermicompost were largely composed by their own or exclusive ASVs (Fig. 5, Supplementary Tables S4, S5).

Several studies have found important levels of reduction of microbial human pathogens during vermicomposting of sewage sludge^27–29^ and animal manures^30,31^. We have found that earthworm activity, mainly during the gut-associated processes, is a critical factor leading to the rapid reduction of pathogens during vermicomposting.

The mechanisms involved in the reduction or elimination of the bacterial and fungal taxa in the sewage sludge may include direct effects of physical disruption during grinding in the earthworm gizzard, microbial inhibition by antimicrobial substances or microbial antagonists produced by the earthworms themselves, and destruction of microorganisms by enzymatic digestion and assimilation^15^.

This underscores the critical importance of maintaining vermicomposting reactors at the highest possible stocking densities or at maximum charge capacity, so optimal operation or performance of the vermicomposting process is ensured.

This study describes how vermicomposting drastically modifies bacterial and fungal communities of sewage sludge and stresses the critical effect of earthworms during that process. Bacterial and fungal composition and structure changes significantly during gut-associated processes (GAP) and cast-associated processes (CAP). Most of the microbial taxa present in the sewage sludge were eliminated during vermicomposting, mainly in the GAP. Given that earthworms change drastically microbial communities of the organic wastes during vermicomposting and vermicompost microbiome resembles the microbial communities of the earthworm gut, studying the effect of the starting material in the configuration of the earthworm gut microbiome is paramount.

## Methods

### Sewage sludge, vermicomposting and sampling

Sewage sludge used as vermicomposting feedstock was obtained from a wastewater treatment plant in Caldas de Reis (9,775 inhabitants), Galicia, Northwestern Spain. Raw sewage sludge was processed in medium-scale vermireactors (1 m^2^) housed in the greenhouse facilities of the Animal Ecology Group (GEA) at the University of Vigo (Spain), as described in Domínguez et al.^19^. Briefly, vermicomposting of sewage sludge was carried out in a rectangular plastic pilot-scale vermireactor (1.1 m long × 1.05 m wide × 70 cm high) housed in a greenhouse with no temperature control (Supplementary Fig. S4). Before adding the sewage sludge, the vermireactor contained a layer of vermicompost (12 cm height) as a bed for the earthworms (*Eisenia andrei*). Earthworm population density in the vermireactor was over 12,000 individuals per m2. We added fresh sewage sludge (120 kg fresh weight) to the bed in a 12 cm layer. The vermicompost bedding was separated from fresh sewage sludge by a plastic mesh (5 mm mesh size). Use of the plastic mesh allows earthworm migration, prevents mixing of the processed sludge and the vermicompost bedding and facilitates the sampling of sewage during vermicomposting. The moisture content was maintained at around 85% throughout the duration of the experiment by covering the vermireactor with a shade cloth.

To collect fresh cast samples, i.e. those due to gut associated processes (GAP), adult individuals of the earthworm species *Eisenia andrei* were removed from the vermireactors, washed three times with sterile distilled water and placed in clean, sterile Petri dishes on moistened sterile filter paper (20 individuals per dish, 5 dishes) (Supplementary Fig. S4). Sampling dishes were placed in an incubation chamber in darkness for 24 h. After that, fresh earthworm casts were collected from each sampling dish with a sterile spatula (flame sterilized between samples). Casts were then stored in 1.5 mL Eppendorf tubes at − 80 °C. Vermicompost samples (n = 5), i.e. those due to cast associated processes (CAP), were collected from the vermireactor after 3 months of vermicomposting (Supplementary Fig. S4).

### 16S and ITS rRNA amplification, sequencing and analysis

DNA was extracted from 0.25 g (fresh weight) of each sample (sewage sludge, earthworm casts and vermicompost) using the MO-BIO PowerSoil kit following the manufacturer's protocols. DNA quality and quantity were determined using BioTek’s Take3 Multi-Volume Plate. All laboratory procedures were performed in a laminar flow hood to prevent contamination of the samples with microorganisms from the surrounding environment.

### 16S and ITS rRNA amplification, sequencing and analysis

We amplified and sequenced fragments of the 16S (250 bp) and ITS (250 bp) rRNA genes following the Earth Microbiome Project protocols (https://www.protocols.io/workspaces/earth-microbiome-project). Amplicon libraries were created using primers for the V4 region of the 16S rRNA gene (forward GTGYCAGCMGCCGCGGTAA and reverse GGACTACNVGGGTWTCTAAT) and a fragment of the ITS rRNA gene (ITS1f. forward primer (CTTGGTCATTTAGAGGAAGTAA) and ITS2 reverse primer (GCTGCGTTCTTCATCGATGC)). Amplicon sequencing was done on an Illumina MiSeq genome sequencer at the *Argonne National Laboratory.* DADA2 (version 1.16) was used to infer amplicon sequence variants (ASVs) present in each sample^32^. Exact sequence variants provide a more accurate and reproducible description of amplicon-sequenced communities than is possible with OTUs defined at a constant level (97% or other) of sequence similarity^33^. Bioinformatics processing largely followed the DADA2 pipeline tutorials (https://benjjneb.github.io/dada2/tutorial.html). 16S forward/reverse read pairs were trimmed and filtered, with forward reads truncated at 140 nt and reverse reads at 130 nt, no ambiguous bases allowed, and each read required to have less than two expected errors based on their quality scores. ASVs were independently inferred from the forward and reverse of each sample using the run-specific error rates, and then read pairs were merged. Chimeras were identified in each sample and ASVs were removed if identified as chimeric in a sufficient fraction of the samples in which they were present. We processed ITS reads similarly, but we did not trim them. We also inferred ASVs only from forward reads because taxonomic classification from merged pairs and reverse reads included more uncertainty (10% versus 21% of unclassified reads at phylum level, respectively). Taxonomic assignment was performed against the Silva (version 138) and UNITE (version 8.2) databases for 16S and ITS, respectively, and using the RDP naive Bayesian classifier implemented in the DADA2 R package (min boot 80^34,35^). We discarded ASVs unclassified at phylum level for both 16S (0.6% of sequences) and ITS (4% of sequences). For 16S a total of 100,905 sequences (mean: 6727, SD: 1557) passed all quality filters and were assigned to 1614 ASVs. For ITS a total of 191,011 sequences (mean: 12734, SD: 2573) passed all quality filters and were assigned to 824 ASVs. Rarefaction curves indicated that sampling depth was optimal for all samples (Supplementary Fig. S3).

### Statistical analysis

We analysed and plotted all the data using the phyloseq^36^ and ggplot2^37^ packages implemented in R version 4.0.3^38^.

We studied differential abundance of ASV from bacterial and fungal phyla and genera of sludge before and after transit through the gut of *Eisenia andrei* using negative binomial models as implemented in the package DESeq2^39^. Differential abundances of ASVs and other bacterial and fungal taxa were determined according to Wald tests and p-values adjusted by false discovery rate (p-adj < 0.05). Because multiple pairwise Wald tests were conducted for each pairwise comparison between treatments, we further adjusted “raw” P values using the Benjamini–Hochberg method to correct for multiple pairwise comparisons. After correction, non-significant contrasts were considered to have an effect size (log2 fold change) of zero.

Taxonomic α-diversity was calculated as the number of observed ASVs, and diversity and richness were estimated with the inverse Simpson and Chao1 indices, respectively. Phylogenetic diversity was calculated as Faith’s phylogenetic diversity^40^. The impact of earthworm gut transit on both taxonomic and phylogenetic α-diversity of bacterial and fungal communities of sewage sludge was assessed using non-parametric Kruskal–Wallis tests. We used paired Wilcoxon test for post-hoc comparisons, with Benjamini–Hochberg FDR for multiple test correction.

Taxonomic β-diversity at the ASV level for bacterial and fungal communities was estimated as the difference in the composition of the bacterial taxonomic community among samples of sludge, cast and aged casts. This was done by coupling principal coordinate analysis (PCoA) with distance matrices that take the abundance of ASVs into account (Bray–Curtis) or not (Jaccard). Phylogenetic β-diversity was also estimated by PCoA of weighted (considering abundance of ASVs) and unweighted Unifrac matrix distances^41^. As before, we analyzed differences in β-diversity of bacterial and fungal communities using non-parametric Kruskal–Wallis tests. We also used the paired Wilcoxon test for post-hoc comparisons, with Benjamini–Hochberg FDR for multiple test correction.

We estimated the absolute number of bacterial and fungal ASVs present in sewage sludge, fresh earthworm casts and vermicompost after removing ASVs shared among treatments; hence we consider native sludge ASVs those present only in sludge samples. We estimated shared ASVs among treatments as those ASVs present in pairwise comparisons between treatments.

### Statement

Permission was obtained from the wastewater treatment plant in Caldas de Reis for collection of the sample.

## Supplementary Information

Supplementary Figures.

Supplementary Tables.

## Data Availability

The sequence data generated in the current study are available in the National Center for Biotechnology Information Sequence Read Archive (SRA) under the SRA accession numbers PRJNA723448 for bacteria and PRJNA723452 for fungi.
