# Supplementary material for: Earthworms drastically change fungal and bacterial communities during vermicomposting of sewage sludge
Source: Sci Rep. 2021 Jul 30;11:15556. doi: 10.1038/s41598-021-95099-z (PMC8324770; doi:10.1038/s41598-021-95099-z)
Supplement: Supplementary file 1 — Supplementary Figures. [file 41598_2021_95099_MOESM1_ESM.pdf]

Earthworms drastically change fungal and bacterial communities during vermicomposting of sewage sludge

Jorge Domínguez, Manuel Aira, Keith A. Crandall, Marcos Pérez-Losada

Supplementary Figures

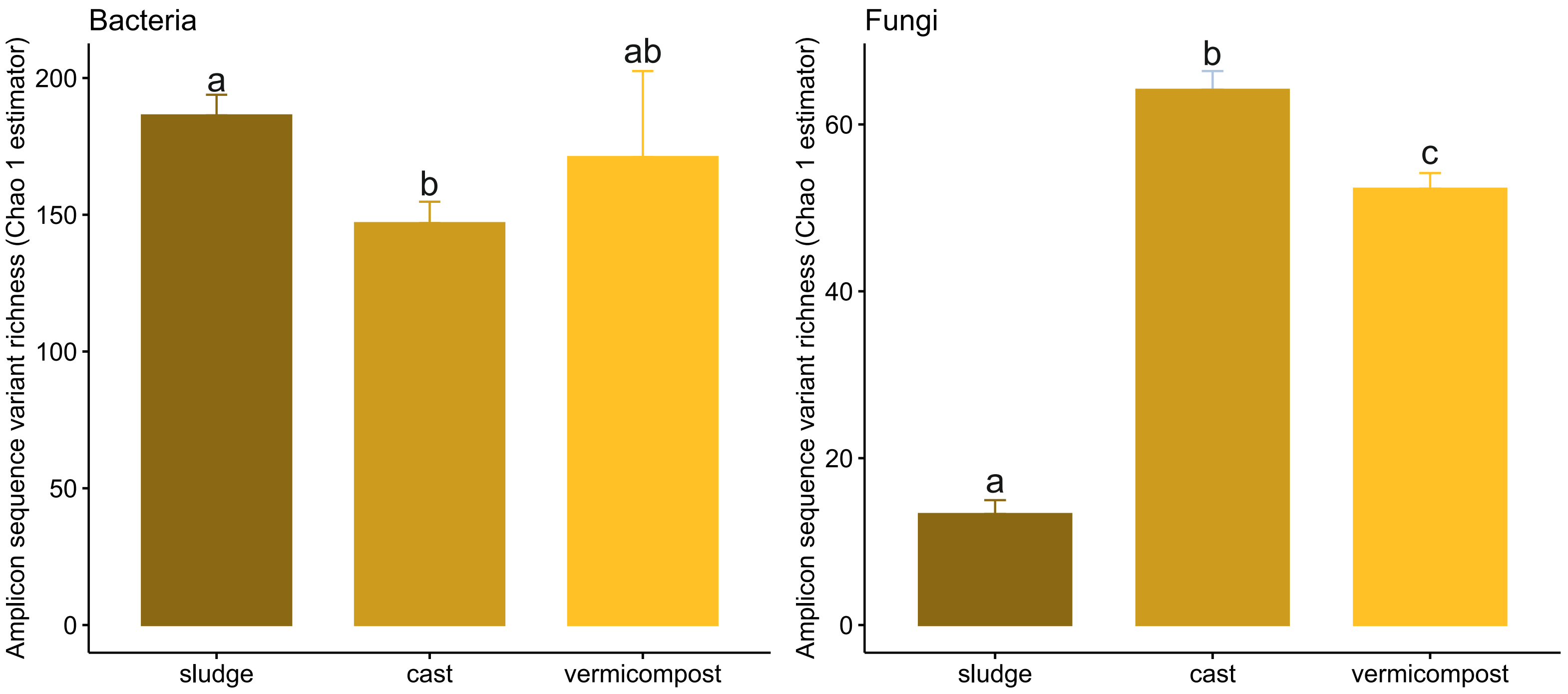

Figure S1: Changes in α-diversity of bacterial and fungal communities during vermicomposting of sewage sludge denoted by Chao 1 richness estimator.

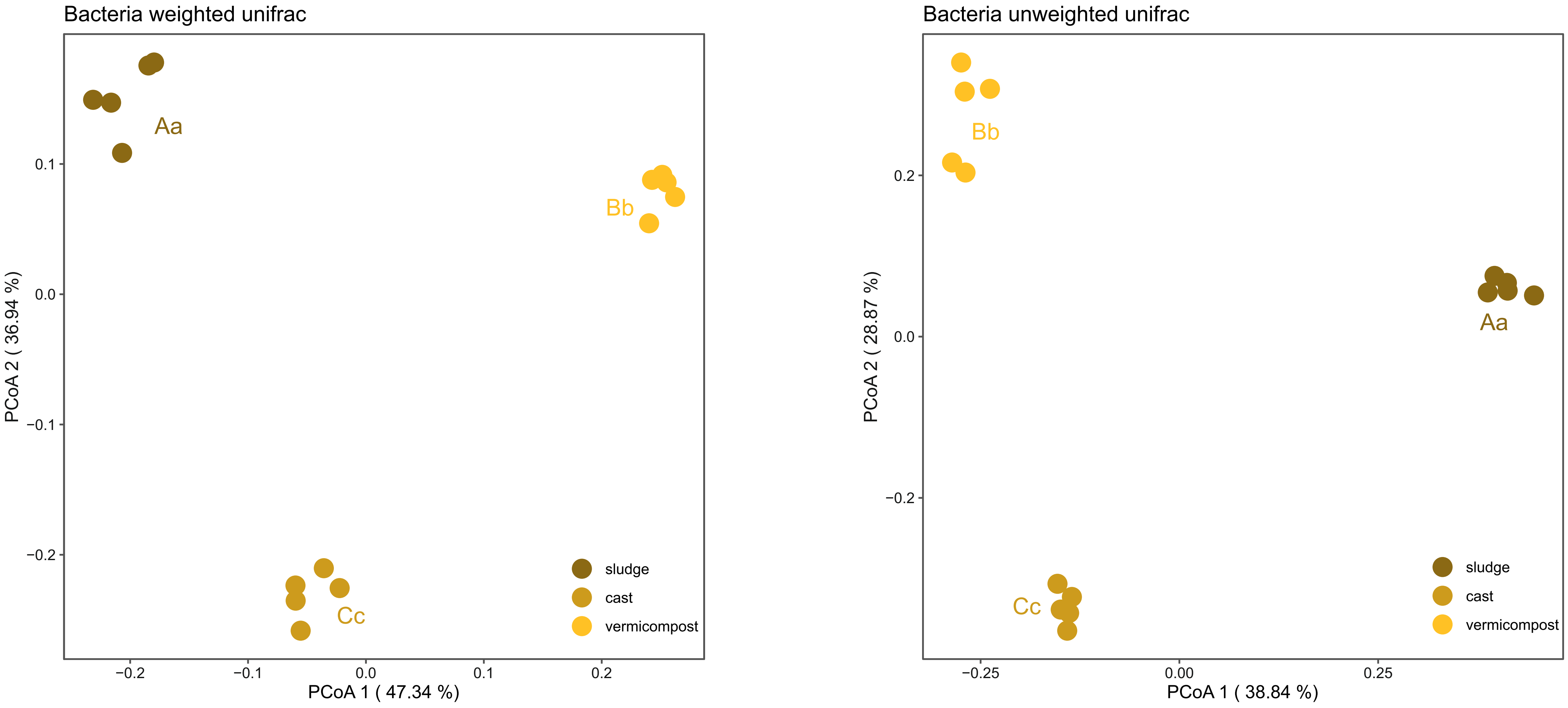

Figure S2: Changes in β-diversity of bacterial communities during vermicomposting of sewage sludge. Principal coordinate analysis with weighted and unweighted UniFrac distances. Different capital and lower-case letters indicate significant differences between sewage sludge, fresh earthworm casts and vermicompost in PCoA 1 and PCoA 2 scores respectively (paired Wilcoxon test, FDR corrected).

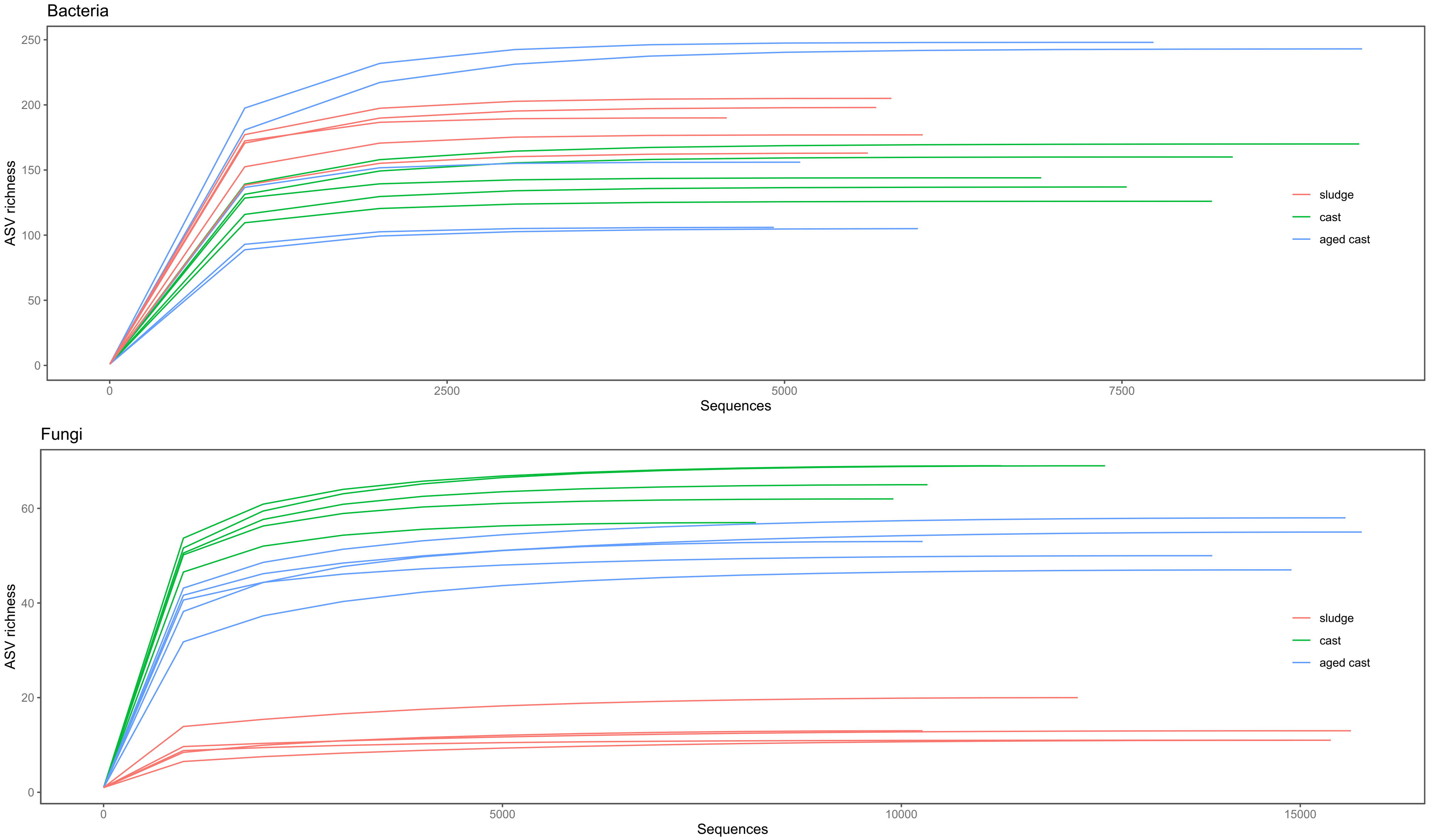

Figure S3: Rarefaction curves indicating the number of amplicon sequence variants (ASVs) of bacteria and fungi found in each sample of sewage sludge and earthworm casts and aged casts.

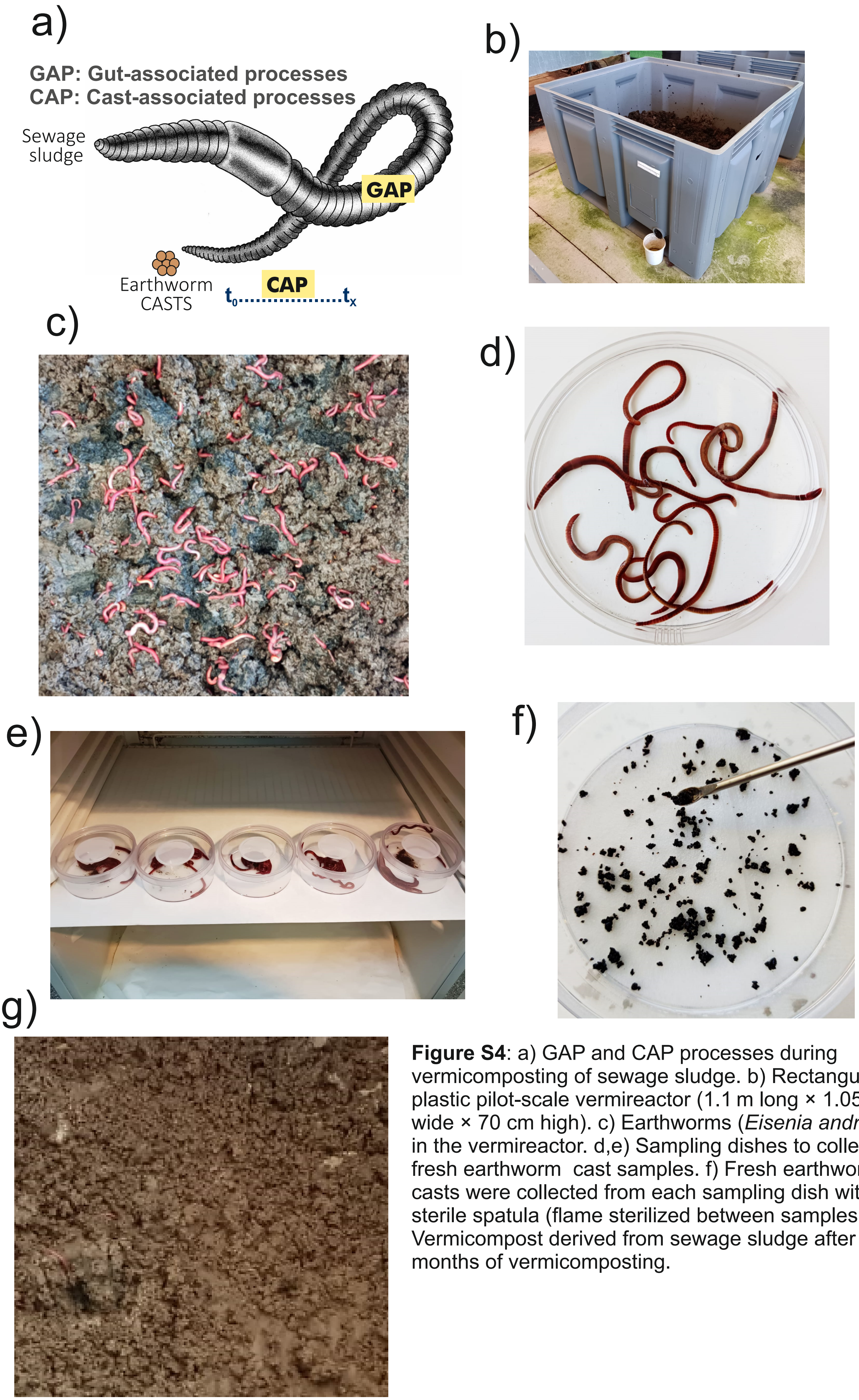

Figure S4: a) GAP and CAP processes during vermicomposting of sewage sludge. b) Rectangular plastic pilot-scale vermireactor (1.1 m long × 1.05 m wide × 70 cm high). c) Earthworms (*Eisenia andrei*) in the vermireactor. d,e) Sampling dishes to collect fresh earthworm cast samples. f) Fresh earthworm casts were collected from each sampling dish with a sterile spatula (flame sterilized between samples). g) Vermicompost derived from sewage sludge after 3 months of vermicomposting.
